# Di-μ-acetato-κ^4^ *O*:*O*-bis­({*N*′-[(*E*)-phen­yl­(pyridin-2-yl-κ*N*)methyl­idene]benzo­hydrazidato-κ^2^ *N*′,*O*}copper(II))

**DOI:** 10.1107/S1600536812031467

**Published:** 2012-07-18

**Authors:** M. C. Vineetha, M. Sithambaresan, Jinsa Mary Jacob, M. R. Prathapachandra Kurup

**Affiliations:** a Department of Applied Chemistry, Cochin University of Science and Technology, Kochi 682 022, India; b Department of Chemistry, Faculty of Science, Eastern University, Sri Lanka, Chenkalady, Sri Lanka

## Abstract

The binuclear molecule of the title compound, [Cu_2_(C_19_H_14_N_3_O)_2_(CH_3_COO)_2_], resides on a crystallographic inversion centre. It has an *E* conformation with respect to the azomethine double bond and a *Z* conformation about the amide C=N bond. The Cu^II^ atom has a slightly distorted square-pyramidal coordination geometry. The crystal packing involves inter­molecular C—H⋯O, C—H⋯N and C—H⋯π and two types of π–π inter­actions, with centroid–centroid distances of 3.9958 (10) and 3.7016 (13) Å.

## Related literature

For the applications of benzohydrazide compounds, see: El-Sayed *et al.* (2011 ▶); Bakir & Brown (2002 ▶). For similar structures, see: Mangalam & Kurup (2011 ▶). For the synthesis of related compounds, see: Mangalam *et al.* (2010 ▶).
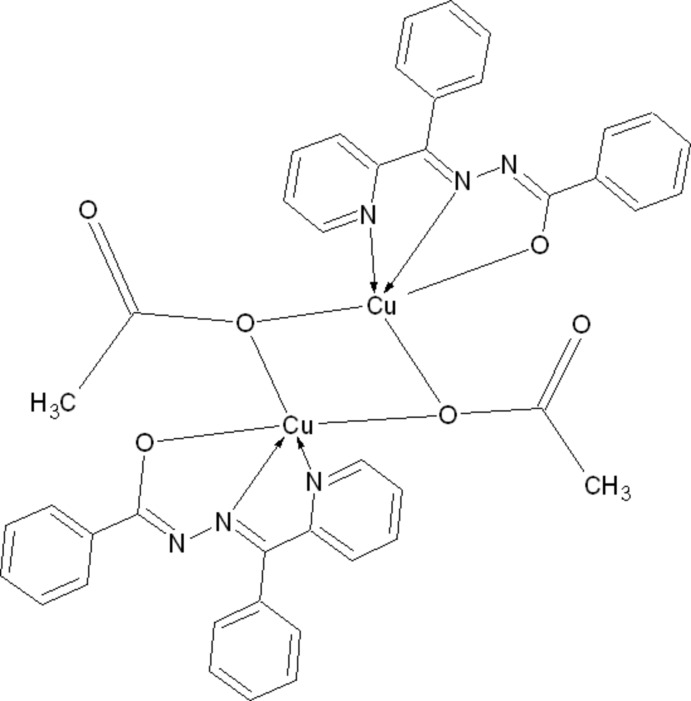


## Experimental

### 

#### Crystal data

[Cu_2_(C_19_H_14_N_3_O)_2_(C_2_H_3_O_2_)_2_]
*M* *_r_* = 845.85
Monoclinic, 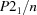

*a* = 9.5758 (3) Å
*b* = 13.1009 (4) Å
*c* = 15.2124 (5) Å
β = 100.718 (1)°
*V* = 1875.13 (10) Å^3^
*Z* = 2
Mo *K*α radiation
μ = 1.19 mm^−1^
*T* = 296 K
0.35 × 0.25 × 0.20 mm

#### Data collection

Bruker Kappa APEXII CCD diffractometer
Absorption correction: multi-scan (*SADABS*; Bruker, 2004 ▶) *T*_min_ = 0.706, *T*_max_ = 0.788
14426 measured reflections
3300 independent reflections
2981 reflections with *I* > 2σ(*I*)
*R*_int_ = 0.022

#### Refinement

*R*[*F* ^2^ > 2σ(*F* ^2^)] = 0.027
*wR*(*F* ^2^) = 0.079
*S* = 1.06
3300 reflections
254 parameters
H-atom parameters constrained
Δρ_max_ = 0.42 e Å^−3^
Δρ_min_ = −0.35 e Å^−3^

### 

Data collection: *APEX2* (Bruker, 2004 ▶); cell refinement: *APEX2* and *SAINT* (Bruker, 2004 ▶); data reduction: *SAINT* and *XPREP* (Bruker, 2004 ▶); program(s) used to solve structure: *SHELXS97* (Sheldrick, 2008 ▶); program(s) used to refine structure: *SHELXL97* (Sheldrick, 2008 ▶); molecular graphics: *ORTEP-3* (Farrugia, 1997 ▶) and *DIAMOND* (Brandenburg, 2010 ▶); software used to prepare material for publication: *SHELXL97* and *publCIF* (Westrip, 2010 ▶).

## Supplementary Material

Crystal structure: contains datablock(s) I, global. DOI: 10.1107/S1600536812031467/fj2577sup1.cif

Structure factors: contains datablock(s) I. DOI: 10.1107/S1600536812031467/fj2577Isup2.hkl

Additional supplementary materials:  crystallographic information; 3D view; checkCIF report

## Figures and Tables

**Table 1:** Hydrogen-bond geometry (Å, °) *Cg*2, *Cg*3, *Cg*5, *Cg*8 and *Cg*9 are the centroids of the Cu1/O1/C13/N3/N2, Cu1/O3/Cu1A/O3A, Cu1/N1/C5/C6/N2, C7–C12 and C14–C19 rings, respectively.

*D*—H⋯*A*	*D*—H	H⋯*A*	*D*⋯*A*	*D*—H⋯*A*
C15—H15⋯N3	0.93	2.43	2.752 (3)	101
C8—H8⋯O1^i^	0.93	2.51	3.416 (2)	166
C15—H15⋯O2^i^	0.93	2.37	3.116 (4)	137
C1—H1⋯*Cg*3	0.93	2.85	3.1701	102
C3—H3⋯*Cg*8^ii^	0.93	2.86	3.5543	132
C12—H12⋯*Cg*9^ii^	0.93	3.12	3.8426	136
C21—H21*A*⋯*Cg*5^iii^	0.96	3.14	3.5809	110
C21—H21*B*⋯*Cg*2^iii^	0.96	2.96	3.6761	133

Symmetry codes: (i) 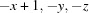; (ii) 
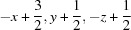
; (iii) 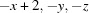.

**Fig. 1.**
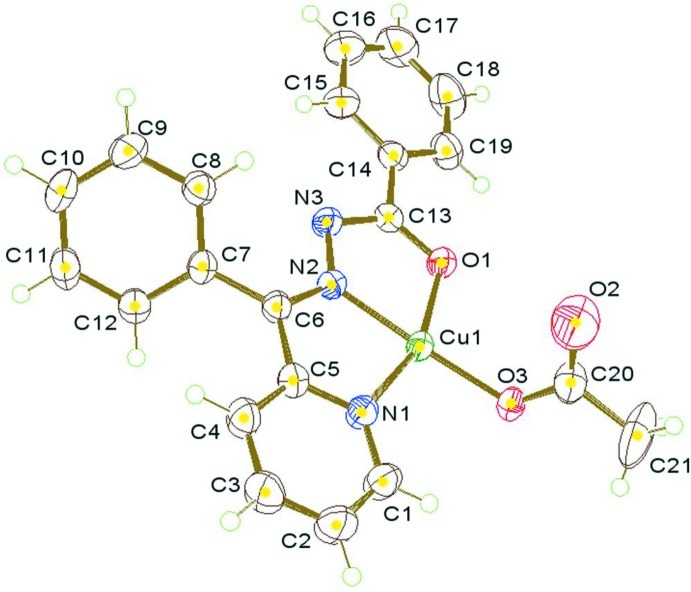
ORTEP view of the unique part of the Cu complex, drawn with 50% probability displacement ellipsoids for the non-H atoms.

**Fig. 2.**
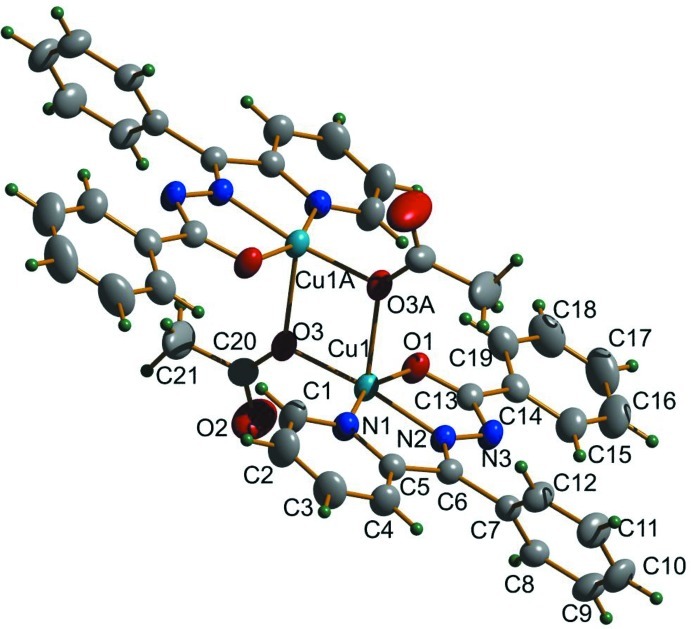
Molecular structure of the title compound.

**Fig. 3.**
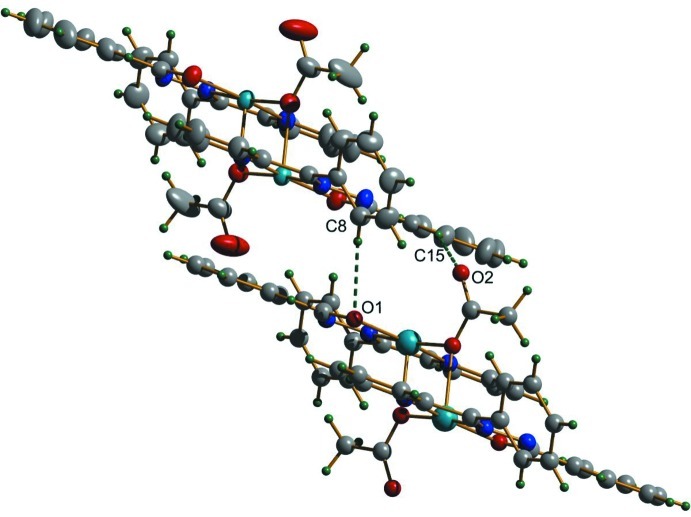
Hydrogen-bonding interactions showing an infinite chain in the crystal structure of [Cu2N6O6C42H34].

**Fig. 4.**
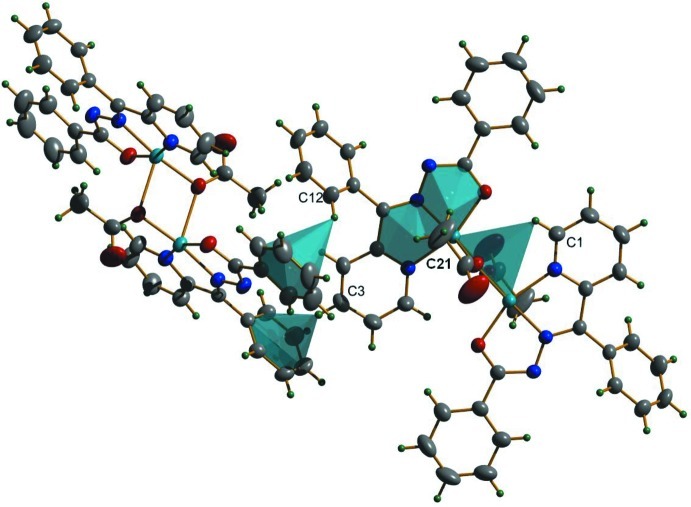
C—H···π interactions found in the title compound.

**Fig. 5.**
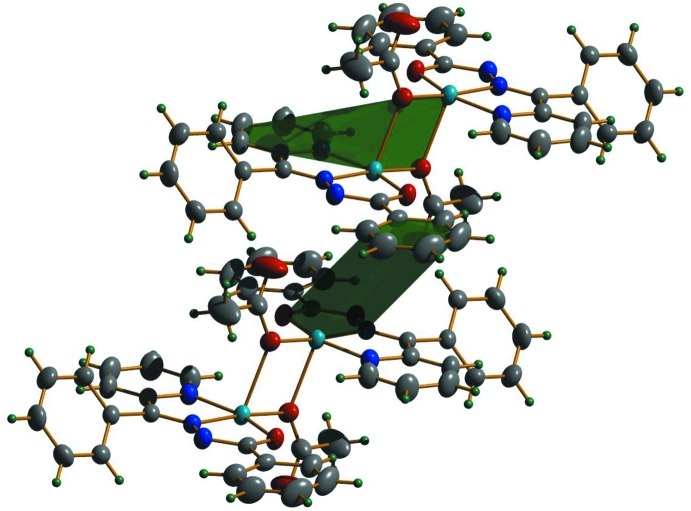
π–π interactions present in the crystal structure of [Cu2N6O6C42H34].

**Fig. 6.**
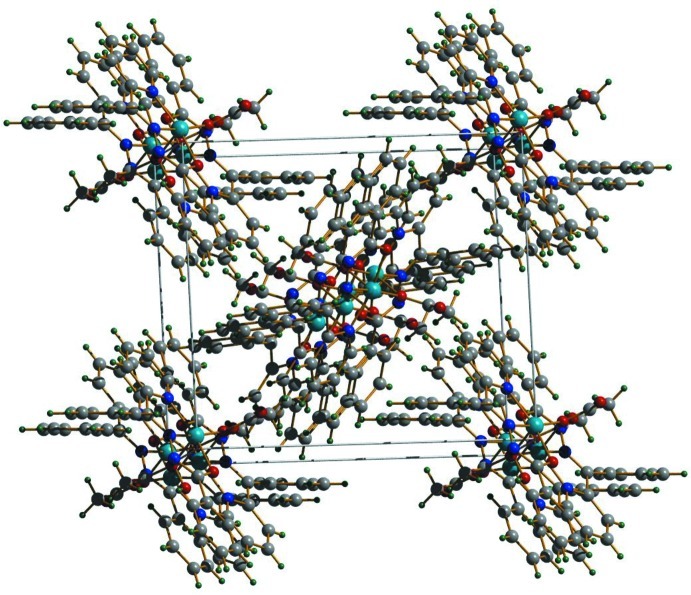
Packing diagram of the compound [Cu2N6O6C42H34] along a axis.

## supplementary crystallographic information

### Comment

Derivatives of benzohydrazides and their metal complexes possess pronounced biological activities. They also have versatile binding properties and show inhibitory activity against ovine COX-2 (El-Sayed *et al.*, 2011). Derivatives of benzohydrazides and their metal complexes have received considerable attention during the last decade because of their versatile applications in nonlinear optics and molecular sensing (Bakir & Brown, 2002).The present report is an extension of our earlier studies in this area (Mangalam & Kurup, 2011).

The compound crystallizes in monoclinic space group *P*2_1_/*n*. The labeled diagrams of the asymmetric unit and the dimeric molecule are shown in Figs. 1 and 2 respectively. This molecule adopts an *E* configuration with respect to C6—N2 bond and it exists in *enolate* form with C13—O1 bond length of 1.276 (2) Å which is very close to a formal C—O bond length [1.31 Å]. The dihedral angle between pyridine and the phenyl (comprising atoms C14—C19) rings is 4.78 (11)°. O1 and N2 are in *Z* configuration with respect to C13—N3 bond having a torsional angle of 1.6 (3)°.

A non-conventional intermolecular hydrogen bond (Fig. 3) is present in the molecular system between the H atoms attached to the C8, C15 atoms and O1, O2 aoms of another molecule with D···A distances of 3.416 (2) and 3.116 (3) Å respectively (Table 1). Moreover, there are C–H···π interactions between the H atoms attached at the C1, C3, C12 and C21 atoms and the corresponding aromatic and metal chelate rings of the same or another molecule (Fig. 4) with the minimum distance of 3.170 (2) Å between the carbon atoms and the corresponding rings involving interactions. There are two types of π–π interactions within the dimeric molecule (T-shaped arrangement) and also between the adjacent molecules (slipped arrangement) with the centroid-centroid distances of 3.9958 (10) and 3.7016 (13) Å respectively between the rings involving interactions as shown in Fig. 5.

Packing of molecules (Fig. 6) is predominantly favored by two types of non-classical intermolecular hydrogen bonding and C–H···π interactions involving the H atoms from C3 and C12 atoms and a π–π interaction between the adjacent molecules in slipped arrangement.

### Experimental

The title complex was prepared by adapting a reported procedure (Mangalam *et al.*, 2010) by refluxing a mixture of methanolic solutions of *N'*-[(*E*)-phenyl(pyridin-2-yl)methylidene]benzohydrazide (0.301 g, 1 mmol) and Cu(OAc)_2_.H_2_O (0.199 g, 1 mmol) for three hours. After two days, green colored crystals were collected, washed with few drops of methanol and dried over P_4_O_10_*in vacuo*. Single crystals of the title complex suitable for X-ray analysis were obtained after two days from the mother liquor by slow evaporation.

### Refinement

All H atoms on C were placed in calculated positions, guided by difference maps, with C—H bond distance of 0.93–0.96 Å. H atoms were assigned as *U*_iso_=1.2Ueq (1.5 for Me).

### Crystal data

**Table:**

[Cu_2_(C_19_H_14_N_3_O)_2_(C_2_H_3_O_2_)_2_]	*F*(000) = 868.0
*M**_r_* = 845.85	*D*_x_ = 1.498 Mg m^−^^3^
Monoclinic, *P*2_1_/*n*	Mo *K*α radiation, λ = 0.71073 Å
Hall symbol: -P 2yn	Cell parameters from 9946 reflections
*a* = 9.5758 (3) Å	θ = 2.7–28.3°
*b* = 13.1009 (4) Å	µ = 1.19 mm^−^^1^
*c* = 15.2124 (5) Å	*T* = 296 K
β = 100.718 (1)°	Block, green
*V* = 1875.13 (10) Å^3^	0.35 × 0.25 × 0.20 mm
*Z* = 2	

### Data collection

**Table:**

Bruker Kappa APEXII CCD diffractometer	3300 independent reflections
Radiation source: fine-focus sealed tube	2981 reflections with *I* > 2σ(*I*)
Graphite monochromator	*R*_int_ = 0.022
ω and φ scan	θ_max_ = 25.0°, θ_min_ = 2.7°
Absorption correction: multi-scan (*SADABS*; Bruker, 2004)	*h* = −11→11
*T*_min_ = 0.706, *T*_max_ = 0.788	*k* = −15→14
14426 measured reflections	*l* = −18→17

### Refinement

**Table:**

Refinement on *F*^2^	Primary atom site location: structure-invariant direct methods
Least-squares matrix: full	Secondary atom site location: difference Fourier map
*R*[*F*^2^ > 2σ(*F*^2^)] = 0.027	Hydrogen site location: inferred from neighbouring sites
*wR*(*F*^2^) = 0.079	H-atom parameters constrained
*S* = 1.06	*w* = 1/[σ^2^(*F*_o_^2^) + (0.0437*P*)^2^ + 0.9597*P*] where *P* = (*F*_o_^2^ + 2*F*_c_^2^)/3
3300 reflections	(Δ/σ)_max_ = 0.001
254 parameters	Δρ_max_ = 0.42 e Å^−^^3^
0 restraints	Δρ_min_ = −0.35 e Å^−^^3^

### Special details

**Table:**

Geometry. All e.s.d.'s (except the e.s.d. in the dihedral angle between two l.s. planes) are estimated using the full covariance matrix. The cell e.s.d.'s are taken into account individually in the estimation of e.s.d.'s in distances, angles and torsion angles; correlations between e.s.d.'s in cell parameters are only used when they are defined by crystal symmetry. An approximate (isotropic) treatment of cell e.s.d.'s is used for estimating e.s.d.'s involving l.s. planes.
Refinement. Refinement of *F*^2^ against ALL reflections. The weighted *R*-factor *wR* and goodness of fit *S* are based on *F*^2^, conventional *R*-factors *R* are based on *F*, with *F* set to zero for negative *F*^2^. The threshold expression of *F*^2^ > σ(*F*^2^) is used only for calculating *R*-factors(gt) *etc*. and is not relevant to the choice of reflections for refinement. *R*-factors based on *F*^2^ are statistically about twice as large as those based on *F*, and *R*- factors based on ALL data will be even larger.

### Fractional atomic coordinates and isotropic or equivalent isotropic displacement parameters (Å^2^)

**Table:**

	*x*	*y*	*z*	*U*_iso_*/*U*_eq_	
Cu1	0.83940 (2)	0.033872 (17)	0.018513 (14)	0.03149 (10)	
O1	0.72812 (15)	−0.09187 (11)	−0.01867 (9)	0.0371 (3)	
O2	0.7660 (2)	0.1008 (2)	−0.17172 (15)	0.1028 (9)	
N1	0.90507 (17)	0.16141 (13)	0.08915 (11)	0.0357 (4)	
N2	0.71446 (17)	0.03165 (11)	0.10470 (11)	0.0308 (3)	
N3	0.62458 (18)	−0.04952 (12)	0.10243 (11)	0.0345 (4)	
O3	0.95034 (15)	0.05070 (11)	−0.07436 (9)	0.0379 (3)	
C1	1.0076 (2)	0.22526 (17)	0.07683 (15)	0.0463 (5)	
H1	1.0587	0.2115	0.0318	0.056*	
C2	1.0406 (3)	0.31125 (19)	0.12869 (17)	0.0547 (6)	
H2	1.1134	0.3544	0.1191	0.066*	
C3	0.9649 (3)	0.33231 (18)	0.19451 (16)	0.0524 (6)	
H3	0.9848	0.3905	0.2296	0.063*	
C4	0.8588 (2)	0.26633 (16)	0.20825 (14)	0.0428 (5)	
H4	0.8061	0.2797	0.2525	0.051*	
C5	0.8318 (2)	0.18018 (15)	0.15558 (12)	0.0334 (4)	
C6	0.7253 (2)	0.10156 (14)	0.16553 (12)	0.0312 (4)	
C7	0.6441 (2)	0.10292 (14)	0.23916 (12)	0.0322 (4)	
C8	0.4970 (2)	0.09464 (15)	0.22120 (13)	0.0370 (4)	
H8	0.4489	0.0884	0.1625	0.044*	
C9	0.4224 (2)	0.09563 (18)	0.29038 (15)	0.0476 (5)	
H9	0.3237	0.0916	0.2779	0.057*	
C10	0.4924 (3)	0.1025 (2)	0.37747 (16)	0.0533 (6)	
H10	0.4415	0.1025	0.4239	0.064*	
C11	0.6387 (3)	0.1093 (2)	0.39599 (14)	0.0519 (6)	
H11	0.6863	0.1132	0.4550	0.062*	
C12	0.7147 (2)	0.11039 (18)	0.32757 (14)	0.0428 (5)	
H12	0.8132	0.1161	0.3404	0.051*	
C13	0.6408 (2)	−0.10829 (15)	0.03375 (12)	0.0323 (4)	
C14	0.5487 (2)	−0.20007 (15)	0.01984 (13)	0.0346 (4)	
C15	0.4417 (2)	−0.21282 (19)	0.06900 (15)	0.0476 (5)	
H15	0.4269	−0.1630	0.1098	0.057*	
C16	0.3573 (3)	−0.2982 (2)	0.05801 (19)	0.0638 (7)	
H16	0.2863	−0.3062	0.0916	0.077*	
C17	0.3777 (3)	−0.3715 (2)	−0.00235 (19)	0.0698 (8)	
H17	0.3210	−0.4296	−0.0095	0.084*	
C18	0.4814 (3)	−0.3593 (2)	−0.05222 (17)	0.0637 (7)	
H18	0.4942	−0.4090	−0.0936	0.076*	
C19	0.5674 (3)	−0.27385 (17)	−0.04190 (14)	0.0463 (5)	
H19	0.6374	−0.2660	−0.0763	0.056*	
C20	0.8914 (2)	0.08509 (18)	−0.15067 (14)	0.0455 (5)	
C21	0.9882 (4)	0.1050 (3)	−0.2152 (2)	0.0952 (13)	
H21A	1.0058	0.0423	−0.2439	0.143*	
H21B	1.0764	0.1323	−0.1836	0.143*	
H21C	0.9445	0.1531	−0.2594	0.143*	

### Atomic displacement parameters (Å^2^)

**Table:**

	*U*^11^	*U*^22^	*U*^33^	*U*^12^	*U*^13^	*U*^23^
Cu1	0.03334 (16)	0.03564 (16)	0.02741 (15)	−0.00451 (9)	0.01064 (10)	−0.00404 (9)
O1	0.0369 (8)	0.0436 (8)	0.0331 (7)	−0.0076 (6)	0.0121 (6)	−0.0094 (6)
O2	0.0589 (13)	0.175 (3)	0.0736 (14)	0.0407 (15)	0.0096 (11)	0.0382 (16)
N1	0.0380 (9)	0.0349 (9)	0.0361 (9)	−0.0038 (7)	0.0123 (7)	−0.0028 (7)
N2	0.0294 (8)	0.0340 (9)	0.0298 (8)	−0.0039 (6)	0.0076 (6)	−0.0032 (6)
N3	0.0346 (9)	0.0362 (9)	0.0342 (9)	−0.0071 (7)	0.0103 (7)	−0.0055 (7)
O3	0.0364 (8)	0.0505 (8)	0.0283 (7)	−0.0005 (6)	0.0098 (6)	0.0041 (6)
C1	0.0494 (13)	0.0432 (12)	0.0513 (13)	−0.0107 (10)	0.0222 (10)	−0.0059 (10)
C2	0.0600 (15)	0.0459 (13)	0.0623 (15)	−0.0207 (11)	0.0217 (12)	−0.0094 (11)
C3	0.0677 (16)	0.0393 (12)	0.0511 (13)	−0.0145 (11)	0.0137 (12)	−0.0130 (10)
C4	0.0517 (13)	0.0404 (11)	0.0385 (11)	−0.0031 (10)	0.0140 (9)	−0.0087 (9)
C5	0.0363 (10)	0.0333 (10)	0.0309 (9)	0.0010 (8)	0.0067 (8)	−0.0010 (8)
C6	0.0322 (10)	0.0334 (10)	0.0284 (9)	0.0017 (8)	0.0066 (7)	−0.0019 (8)
C7	0.0369 (10)	0.0311 (10)	0.0297 (9)	0.0013 (8)	0.0090 (8)	−0.0020 (8)
C8	0.0366 (10)	0.0388 (11)	0.0355 (10)	0.0026 (8)	0.0062 (8)	−0.0025 (8)
C9	0.0370 (11)	0.0556 (14)	0.0529 (13)	0.0042 (10)	0.0156 (10)	−0.0066 (11)
C10	0.0554 (14)	0.0681 (16)	0.0430 (12)	0.0039 (12)	0.0265 (11)	−0.0073 (11)
C11	0.0568 (14)	0.0702 (16)	0.0288 (10)	0.0038 (12)	0.0082 (10)	−0.0053 (10)
C12	0.0365 (11)	0.0575 (13)	0.0344 (11)	0.0018 (10)	0.0066 (9)	−0.0043 (10)
C13	0.0298 (9)	0.0375 (10)	0.0284 (9)	−0.0009 (8)	0.0021 (7)	−0.0005 (8)
C14	0.0340 (10)	0.0360 (10)	0.0312 (9)	−0.0028 (8)	−0.0005 (8)	0.0013 (8)
C15	0.0419 (12)	0.0531 (13)	0.0479 (12)	−0.0103 (10)	0.0087 (10)	−0.0026 (10)
C16	0.0564 (15)	0.0699 (18)	0.0658 (17)	−0.0264 (13)	0.0132 (13)	0.0007 (14)
C17	0.081 (2)	0.0578 (16)	0.0663 (17)	−0.0349 (15)	0.0025 (15)	0.0036 (14)
C18	0.094 (2)	0.0429 (13)	0.0508 (14)	−0.0153 (13)	0.0047 (14)	−0.0101 (11)
C19	0.0582 (14)	0.0409 (12)	0.0388 (11)	−0.0057 (10)	0.0062 (10)	−0.0021 (9)
C20	0.0477 (13)	0.0539 (13)	0.0363 (11)	0.0141 (11)	0.0119 (10)	0.0058 (10)
C21	0.100 (2)	0.138 (3)	0.0595 (18)	0.051 (2)	0.0444 (17)	0.054 (2)

### Geometric parameters (Å, º)

**Table:**

Cu1—O3	1.9318 (14)	C8—C9	1.378 (3)
Cu1—N2	1.9325 (16)	C8—H8	0.9300
Cu1—O1	1.9862 (14)	C9—C10	1.372 (3)
Cu1—N1	2.0225 (16)	C9—H9	0.9300
Cu1—O3^i^	2.3167 (15)	C10—C11	1.379 (3)
O1—C13	1.276 (2)	C10—H10	0.9300
O2—C20	1.202 (3)	C11—C12	1.376 (3)
N1—C1	1.330 (3)	C11—H11	0.9300
N1—C5	1.356 (2)	C12—H12	0.9300
N2—C6	1.292 (2)	C13—C14	1.483 (3)
N2—N3	1.364 (2)	C14—C19	1.382 (3)
N3—C13	1.330 (2)	C14—C15	1.386 (3)
O3—C20	1.275 (3)	C15—C16	1.371 (3)
O3—Cu1^i^	2.3167 (15)	C15—H15	0.9300
C1—C2	1.378 (3)	C16—C17	1.367 (4)
C1—H1	0.9300	C16—H16	0.9300
C2—C3	1.369 (3)	C17—C18	1.366 (4)
C2—H2	0.9300	C17—H17	0.9300
C3—C4	1.379 (3)	C18—C19	1.381 (3)
C3—H3	0.9300	C18—H18	0.9300
C4—C5	1.381 (3)	C19—H19	0.9300
C4—H4	0.9300	C20—C21	1.492 (3)
C5—C6	1.476 (3)	C21—H21A	0.9600
C6—C7	1.478 (3)	C21—H21B	0.9600
C7—C8	1.388 (3)	C21—H21C	0.9600
C7—C12	1.392 (3)		
			
O3—Cu1—N2	172.74 (6)	C7—C8—H8	120.0
O3—Cu1—O1	103.02 (6)	C10—C9—C8	120.5 (2)
N2—Cu1—O1	79.26 (6)	C10—C9—H9	119.7
O3—Cu1—N1	97.78 (6)	C8—C9—H9	119.7
N2—Cu1—N1	79.78 (6)	C9—C10—C11	119.8 (2)
O1—Cu1—N1	159.04 (6)	C9—C10—H10	120.1
O3—Cu1—O3^i^	76.36 (6)	C11—C10—H10	120.1
N2—Cu1—O3^i^	110.45 (6)	C12—C11—C10	120.4 (2)
O1—Cu1—O3^i^	95.24 (6)	C12—C11—H11	119.8
N1—Cu1—O3^i^	92.13 (6)	C10—C11—H11	119.8
C13—O1—Cu1	109.93 (12)	C11—C12—C7	120.0 (2)
C1—N1—C5	119.29 (17)	C11—C12—H12	120.0
C1—N1—Cu1	127.57 (14)	C7—C12—H12	120.0
C5—N1—Cu1	113.14 (13)	O1—C13—N3	125.39 (18)
C6—N2—N3	122.48 (16)	O1—C13—C14	119.27 (17)
C6—N2—Cu1	119.83 (13)	N3—C13—C14	115.34 (17)
N3—N2—Cu1	117.50 (12)	C19—C14—C15	119.0 (2)
C13—N3—N2	107.80 (15)	C19—C14—C13	121.00 (18)
C20—O3—Cu1	119.72 (14)	C15—C14—C13	120.04 (18)
C20—O3—Cu1^i^	135.26 (13)	C16—C15—C14	120.7 (2)
Cu1—O3—Cu1^i^	103.64 (6)	C16—C15—H15	119.7
N1—C1—C2	122.0 (2)	C14—C15—H15	119.7
N1—C1—H1	119.0	C17—C16—C15	120.0 (3)
C2—C1—H1	119.0	C17—C16—H16	120.0
C3—C2—C1	119.2 (2)	C15—C16—H16	120.0
C3—C2—H2	120.4	C18—C17—C16	120.0 (2)
C1—C2—H2	120.4	C18—C17—H17	120.0
C2—C3—C4	119.3 (2)	C16—C17—H17	120.0
C2—C3—H3	120.3	C17—C18—C19	120.8 (2)
C4—C3—H3	120.3	C17—C18—H18	119.6
C3—C4—C5	119.2 (2)	C19—C18—H18	119.6
C3—C4—H4	120.4	C18—C19—C14	119.6 (2)
C5—C4—H4	120.4	C18—C19—H19	120.2
N1—C5—C4	120.91 (18)	C14—C19—H19	120.2
N1—C5—C6	114.38 (16)	O2—C20—O3	123.7 (2)
C4—C5—C6	124.71 (18)	O2—C20—C21	120.5 (2)
N2—C6—C5	112.72 (16)	O3—C20—C21	115.9 (2)
N2—C6—C7	124.57 (17)	C20—C21—H21A	109.5
C5—C6—C7	122.69 (16)	C20—C21—H21B	109.5
C8—C7—C12	119.23 (18)	H21A—C21—H21B	109.5
C8—C7—C6	120.52 (17)	C20—C21—H21C	109.5
C12—C7—C6	120.24 (18)	H21A—C21—H21C	109.5
C9—C8—C7	120.04 (19)	H21B—C21—H21C	109.5
C9—C8—H8	120.0		
			
O3—Cu1—O1—C13	−175.12 (12)	N3—N2—C6—C7	0.1 (3)
N2—Cu1—O1—C13	−2.16 (13)	Cu1—N2—C6—C7	−174.73 (14)
N1—Cu1—O1—C13	−2.4 (3)	N1—C5—C6—N2	−4.3 (2)
O3^i^—Cu1—O1—C13	107.70 (13)	C4—C5—C6—N2	176.11 (19)
O3—Cu1—N1—C1	−7.55 (19)	N1—C5—C6—C7	174.16 (17)
N2—Cu1—N1—C1	179.4 (2)	C4—C5—C6—C7	−5.4 (3)
O1—Cu1—N1—C1	179.61 (18)	N2—C6—C7—C8	−52.9 (3)
O3^i^—Cu1—N1—C1	68.97 (19)	C5—C6—C7—C8	128.8 (2)
O3—Cu1—N1—C5	172.18 (13)	N2—C6—C7—C12	125.8 (2)
N2—Cu1—N1—C5	−0.90 (13)	C5—C6—C7—C12	−52.5 (3)
O1—Cu1—N1—C5	−0.7 (3)	C12—C7—C8—C9	1.1 (3)
O3^i^—Cu1—N1—C5	−111.30 (14)	C6—C7—C8—C9	179.77 (19)
O1—Cu1—N2—C6	178.37 (16)	C7—C8—C9—C10	−1.5 (3)
N1—Cu1—N2—C6	−1.72 (15)	C8—C9—C10—C11	0.6 (4)
O3^i^—Cu1—N2—C6	86.73 (15)	C9—C10—C11—C12	0.7 (4)
O1—Cu1—N2—N3	3.27 (13)	C10—C11—C12—C7	−1.0 (4)
N1—Cu1—N2—N3	−176.82 (15)	C8—C7—C12—C11	0.1 (3)
O3^i^—Cu1—N2—N3	−88.37 (14)	C6—C7—C12—C11	−178.5 (2)
C6—N2—N3—C13	−178.48 (18)	Cu1—O1—C13—N3	0.9 (2)
Cu1—N2—N3—C13	−3.5 (2)	Cu1—O1—C13—C14	−178.53 (13)
O1—Cu1—O3—C20	76.25 (17)	N2—N3—C13—O1	1.6 (3)
N1—Cu1—O3—C20	−101.13 (17)	N2—N3—C13—C14	−178.92 (15)
O3^i^—Cu1—O3—C20	168.6 (2)	O1—C13—C14—C19	7.6 (3)
O1—Cu1—O3—Cu1^i^	−92.32 (6)	N3—C13—C14—C19	−171.86 (19)
N1—Cu1—O3—Cu1^i^	90.31 (7)	O1—C13—C14—C15	−172.24 (19)
O3^i^—Cu1—O3—Cu1^i^	0.0	N3—C13—C14—C15	8.3 (3)
C5—N1—C1—C2	−1.1 (3)	C19—C14—C15—C16	1.3 (3)
Cu1—N1—C1—C2	178.66 (18)	C13—C14—C15—C16	−178.8 (2)
N1—C1—C2—C3	−0.5 (4)	C14—C15—C16—C17	−0.5 (4)
C1—C2—C3—C4	0.9 (4)	C15—C16—C17—C18	−0.5 (5)
C2—C3—C4—C5	0.3 (4)	C16—C17—C18—C19	0.6 (4)
C1—N1—C5—C4	2.3 (3)	C17—C18—C19—C14	0.3 (4)
Cu1—N1—C5—C4	−177.43 (16)	C15—C14—C19—C18	−1.2 (3)
C1—N1—C5—C6	−177.25 (18)	C13—C14—C19—C18	178.9 (2)
Cu1—N1—C5—C6	3.0 (2)	Cu1—O3—C20—O2	−6.1 (4)
C3—C4—C5—N1	−2.0 (3)	Cu1^i^—O3—C20—O2	158.0 (2)
C3—C4—C5—C6	177.6 (2)	Cu1—O3—C20—C21	174.3 (2)
N3—N2—C6—C5	178.56 (16)	Cu1^i^—O3—C20—C21	−21.6 (4)
Cu1—N2—C6—C5	3.7 (2)		

Symmetry code: (i) −*x*+2, −*y*, −*z*.

### Hydrogen-bond geometry (Å, º)

*Cg*2, *Cg*3, *Cg*5, *Cg*8 and *Cg*9 are the centroids of the Cu1/O1/C13/N3/N2, Cu1/O3/Cu1A/O3A, Cu1/N1/C5/C6/N2, C7–C12 and C14–C19 rings, respectively.

**Table:**

*D*—H···*A*	*D*—H	H···*A*	*D*···*A*	*D*—H···*A*
C15—H15···N3	0.93	2.43	2.752 (3)	101
C8—H8···O1^ii^	0.93	2.51	3.416 (2)	166
C15—H15···O2^ii^	0.93	2.37	3.116 (4)	137
C1—H1···*Cg*3	0.93	2.85	3.1701	102
C3—H3···*Cg*8^iii^	0.93	2.86	3.5543	132
C12—H12···*Cg*9^iii^	0.93	3.12	3.8426	136
C21—H21*A*···*Cg*5^i^	0.96	3.14	3.5809	110
C21—H21*B*···*Cg*2^i^	0.96	2.96	3.6761	133

Symmetry codes: (i) −*x*+2, −*y*, −*z*; (ii) −*x*+1, −*y*, −*z*; (iii) −*x*+3/2, *y*+1/2, −*z*+1/2.
